# Heavy Metal Contamination in Sediments from Wetlands Invaded by *Spartina alterniflora* in the Yellow River Delta

**DOI:** 10.3390/toxics10070374

**Published:** 2022-07-04

**Authors:** Zaiwang Zhang, Tongrui Zhang, Wenhao Yu, Jikun Xu, Jialiang Li, Tao Wu, Suzhe Liu, Haiyang Wang, Yuxia Wang, Shuai Shang, Aiguo Lin

**Affiliations:** 1 Shandong Engineering and Technology Research Center for Ecological Fragile Belt of Yellow River Delta, School of Biological and Environmental Engineering, Binzhou University, Binzhou 256600, China; zzwangbzu@163.com (Z.Z.); 15166178836@163.com (T.Z.); yuwenhao1105@163.com (W.Y.); xjkxiaomai@163.com (J.X.); lijialiangcn@163.com (J.L.); wtsdbz@163.com (T.W.); 2 Management Center of the Yellow River Delta Sustainable Development Research Institute, Dongying 257000, China; wwhhyy20000@163.com (H.W.); wanan45678@126.com (Y.W.); 3 Shandong Anhe Safety Technology Research Institute Company Limited, Binzhou 256600, China; 4 The Second Hydrogeology Engineering Geology Brigade, Shandong Provincial Bureau of Geology and Mineral, Shandong Provincial Lubei Geo-Engineering Exploration Institute, Dezhou 253000, China; liusuzhe1001@163.com

**Keywords:** heavy metal, sediment, *Spartina alterniflora*, Yellow River Delta

## Abstract

Heavy metals are major pollutants that pose threats to wetland environments. In the present study, surface sediments from wetlands vegetated by invasive species *Spartina alterniflora* in the Yellow River Delta were collected and determined for the mass fractions of Co, Ni, As, Cd and Pb. Results showed mass fractions of Co, Ni, As, Cd and Pb in the sediments of the *S. alterniflora* communities ranged from 8.5 to 16.0, 13.9–27.9, 3.2–13.8, 0.08–0.24, and 17.6–37.5 mg/kg dw, respectively, generally presenting an order of Pb > Ni > Co > As > Cd. The levels of heavy metals in sediments in the *S. alterniflora* communities were higher than those in the wetland vegetated by the native plant species *Suaeda heteroptera.* Correlations among metal elements were highly significant, suggesting that they might have the same sources. Clay and TOC were important factors affecting the spatial distribution of metals. The *I_geo_* values of the investigated elements in the sediments were frequently lower than 0, revealing the slight pollution status of these metals. Relatively slight values of *E_r_^i^* and *RI* suggested that the potential ecological risks caused by the 5 metals were low. Our findings could provide a better understanding of the correlation between metal pollution and bio-invasion in wetland ecosystems.

## 1. Introduction

Heavy metals, especially Cd, Pb and Hg, are known as hazardous contaminants that are toxic, undegradable and bioaccumulative and would do harm to living organisms once their dose exceeds certain thresholds [1,2]. They were also described as presumably contaminating elements by the International Union of Pure and Applied Chemistry (IUAPC). Many human activities in industry and agriculture have led to the rising occurrence of these elements in the environment [3]. Environmental and health problems caused by heavy metals have always been topical issues, although they are no longer new/unknown substances to the public [4,5]. Investigations focused on the monitoring and assessment of heavy metals in both the environment and organisms have frequently been conducted on a global scale [6,7,8].

Estuarine regions are commonly densely populated, economically advanced, and industrially developed, but their coast and nearby seas might face severe pollution problems due to some domestic, municipal, industrial, agricultural and/or some other human activities [9]. Wetland is a special ecosystem with many functions and plays an important role in the biogeochemical cycling of a variety of contaminants [10]. Coastal wetlands, such as mangroves, salt marshes and seagrass beds, are essential sinks of both organic and inorganic pollutants, including different trace metals [10,11]. In the wetland ecosystem, sediments store most of the heavy metals and are used to indicate environmental quality [6,9]. The physicochemical properties, such as organic matter and mechanical components of the sediments, are sometimes correlated with the distribution of heavy metals in the wetland [12,13].

The Yellow River Delta (YRD) is well-known for its ever-growing scope and plentiful fresh wetland resources due to the transport of the sediments of the Yellow River in China [14]. The aquatic ecosystems of the Yellow River Estuary are facing great natural interaction between land and sea and intensive human activities of the coastal areas, such as oil mining and refining, aquaculture, agriculture, petrochemical industry and so on [15]. Oilfields and some nearby chemical enterprises put enormous environmental pressure on the wetlands of the YRD [16]. Today, a national strategy for ecological protection and sustainable development of the Yellow River Basin has been formulated and conducted in China. As a consequence, much more attention has been paid to the environmental protection and conservation of wetlands in the YRD area. 

In recent years, the YRD confronted a troublesome problem of the invasion of smooth cordgrass (*Spartina alterniflora*), which expanded rapidly with the distribution area elevated from 40 to 4672 ha between 2008 and 2019 [17,18]. This exotic invasion species could quell native plants and affect the macrobenthic fauna in wetlands [19,20]. Moreover, of course, it could change the physicochemical properties of swamp sediments and may affect the transport and fate of heavy metals [19]. However, the heavy metal contamination in wetlands with the invasion of *S. alterniflora* in YRD is still unknown. Previous investigations on metal pollution in the YRD mainly focus on the marine sediments and/or sediments/soils in native plant species (*Phragmites australis*, *Suaeda salsa*, *Tamarix chinensis,* etc.) communities [9,14,15,20,21]. The contamination status, distribution and ecological risks of metals in the sediments of the *S. alterniflora* haven’t been comprehensively discussed.

In the present study, surface sediments from swamps vegetated by *S. alterniflora* in the Yellow River Delta were collected and detected for the mass fractions of Co, Ni, As Cb and Pb. The main objectives of this investigation were to: (1) determine the levels and distributions of typical heavy metals in sediments in the *S. alterniflora* communities; (2) reveal major factors that affect the distribution of trace elements; and (3) evaluate the potential ecological risks of heavy metals.

## 2. Materials and Methods

### 2.1. Study Areas and Sampling

**Figure 1:**
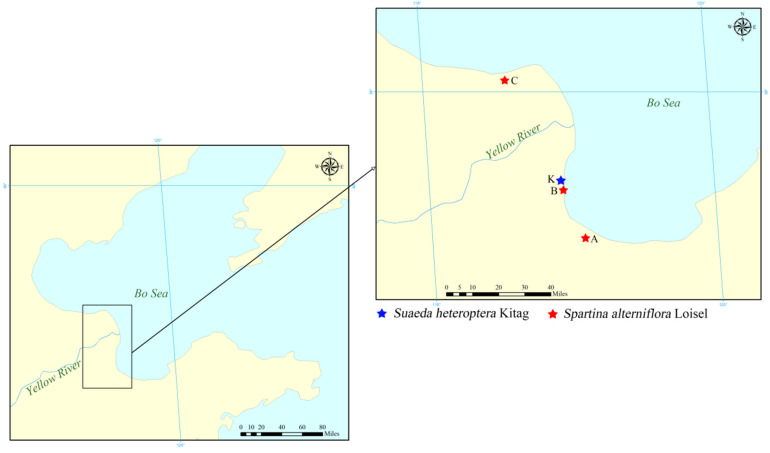
Sampling zones in the Yellow River Delta.

In the present investigation, three major swamps (Zone A, Zone B in Kenli Country, and Zone C in Lijin Country in Dongying City, Shandong Province) vegetated by *S. alterniflora* and one marsh vegetated by native species *S*. *heteroptera* were set as sampling stations (Figure 1). Sampling was conducted in October 2021 and 0–10 cm surface sediment samples (mixed by three sub-samples in a 5 m × 5 m area) were collected using a wooden spade when the tide was out. Each sample was placed in a clean PE package and stored at −20 °C until further analysis.

### 2.2. Sample Analysis

In the laboratory, sediment samples were frozen-dried, ground and passed through a 0.5 mm sieve. Approximately 0.15 g sediment samples were transferred into the digestion vessel, adding a mixture of 6 mL nitric acid (65%), 2 mL hydrochloric acid (37%) and 2 mL hydrofluoric acid (40%), and digested at 190 °C for 30 min using a high-performance microwave digestion system (Ethos Up, Milestone, Sorisole, Italy). Then, evaporation was conducted using electrothermal treatment. The digested samples were diluted to a specified volume by adding 5% HNO_3_. After filtration, heavy metals were determined for Co, Ni, As, Cd and Pb using an inductively coupled plasma mass spectrometer (iCAP RQ, Thermo Scientific, Bremen, Germany). Mass fractions of metal elements are presented in mg/kg dry weight (dw).

Physicochemical properties including total organic carbon (TOC), particle size and pH of the sediments were determined according to the related national standards (GB 17378-2007). TOC was measured using the potassium chromate volumetric analysis method. In short, dried samples were first digested using potassium bichromate and then titrated with ferrous ammonium sulfate. A pH meter was employed to measure the pH values in 1:5 soil-water mixtures. Particle size was determined using the densimeter method. In short, the sediment samples were first frozen-dried and crushed. Then, the samples were treated with H_2_O_2_ (30%) and HCl (1 mol/L) to remove organic matter and carbonates. After that, the samples were suspended in water, and sodium hexametaphosphate solution was added as a dispersant. Then, the samples were ground to several meshes (with sizes of 2 mm, 0.60 mm, 0.212 mm and 63 μm, respectively). Those with a particle size <63 μm were determined using a pipette method through pipetting suspension at different points in time and measuring the weight of the dried particles.

Quality control measures in the metal determination processes included analysis of reagent blank, sample blank, reference materials (ERM-S-510204, TMRM, Changzhou, China), and duplicate samples. The recoveries of the reference materials ranged from 76% to 95%. All glassware and equipment were acid washed with 10% HNO_3_ and rinsed with double-distilled water to avoid possible metal contamination.

### 2.3. Statistical Analysis

Statistical analysis was performed with SPSS 19.0 (SPSS Inc., Chicago, IL, USA). A one-way analysis of variance (ANOVA) was used to determine the spatial variances of the metal mass fractions among the different swamps. Principle components analysis (PCA), rotated component matrices and Spearman’s correlation with the two-tailed test were conducted to explore the correlation and potential sources of heavy metals and identify the major influencing factors of metal distribution in the *S. alterniflora* wetlands.

### 2.4. Ecological Risk Assessment Methods

To identify and estimate the potential ecological risks of heavy metals in wetland sediments, two typical indexes, the geoacccumulation index (*I_geo_*) and the potential ecological risk index (*RI*), were calculated.

*I_geo_* was calculated using the equation *I_geo_* = log_2_ (C*_i_*/1.5B*_i_*) [22], where C*_i_* was the concentration of metal *i* detected in wetland sediment at each sampling site. B*_i_* was the background value of metal *i*. In the present study, soil geochemical reference values of Co, Ni, As, Cd and Pb of Dongying City were chosen as the associated background values [23]. 1.5 was a correlation factor representing both natural and human factors. According to the *I_geo_* values, the degrees of contamination were listed as not contaminated (≤0), slightly contaminated (0–1), moderately contaminated (1–2), moderately to highly contaminated (2–3), highly contaminated (3–4), highly to very highly contaminated (4–5) and very highly contaminated (>5) [6].

The potential ecological risk index (*RI*) is commonly used to describe and evaluate the ecological risk degree of heavy metals in sediments, taking the toxicological effects of these trace elements into consideration [24]. The *RI* of an individual metal was calculated using the equations that *E_r_^i^* =*T_r_^i^* × (*C_s_^i^*/*C_n_^i^*), where *C_s_^i^* is the concentration of element *i* in the sediment sample and *C_n_^i^* is the geochemical background value of element *i. E_r_^i^* is the potential ecological risk factor of the heavy metal *i*, *T_r_^i^* is the toxic response factor of element *i*. The *T_r_^i^* values for Co, Ni, As, Cd and Pb are 5, 5, 10, 30 and 5, respectively [25,26]. In addition, the synergistic hazards of multiple metals (*RI*) were calculated as the sum of *E_r_^i^* of each trace element. The degrees of ecological risk of a single metal (*E_r_^i^*) could be listed as five categories, including low (<40), moderate (40–80), considerable (80–160), high (160–320) and very high (>320). The *RI* of multiple metals could be divided into four levels: low (<150), moderate (150–300), high (300–600) and very high (>600).

## 3. Results and Discussion

### 3.1. Levels of Heavy Metals in Sediments

The mass fractions of heavy metals in sediments from different swamps of the Yellow River Delta are presented in Table 1. Mass fractions of Co, Ni, As, Cd and Pb in the sediments of the *S. alterniflora* communities ranged from 8.5 to 16.0, 13.9–27.9, 3.2–13.8, 0.08–0.24 and 17.6–37.5 mg/kg dw, respectively and those in the *S. heteroptera* community were in the range of 7.5–7.9, 11.2–15.5, 5.3–5.8, 0.07–0.08 and 14.7–15.1 mg/kg, respectively. A general order of Pb > Ni > Co > As > Cd (in 93% of the samples) could be observed. This sequence was slightly different from those reported in some other swamps with varied plant species in the Yellow River Delta, where the amounts of Ni tended to be slightly higher than those of Pb [9,26]. The mass fractions of As and Cd were frequently found to be much lower than the other three metals in most previous investigations [9,13].

The background values for Co, Ni, As, Cd and Pb in the soils of Dongying City were 11.4, 27.5, 10.3, 0.13 and 19.4 mg/kg, respectively [23]. By comparing these data, 14.6%, 0%, 9.8%, 2.4% and 17% of the samples were observed with mass fractions over the reference values. In general, our results, including those over-standard values mentioned above, seemed to be close to their related reference objects. These observations indicated that the sediments in both the *S. alterniflora* communities and the *S. heteroptera* community were barely polluted by those metal elements. In recent years, especially after the formulation of the national strategy for ecological protection and sustainable development of the Yellow River Basin of China, great efforts have been made to protect the environment. Much more attention has been paid to the environmental protection and conservation of wetlands in the YRD area. These findings might reveal the achievements of the local government’s environmental protection measures.

Table 2 summarizes the mass fractions of metal elements in sediments from the YRD and some other regions in previous studies. Levels of Co in sediments from wetlands of the YRD seemed to be rarely reported and our results were similar to those reported in *P. australis* wetland [9] and slightly lower than those reported in Luoyuan wetland in China [27] and the fishing zone of Kerala in India [28]. Levels of Ni in the present study were in line with those reported in various types of sediments in the YRD, but lower than those in sediments from the Luoyuan wetland [27], Yangtze River Estuary in China [29] and the fishing zone of Kerala in India [28]. The levels of As in this investigation seemed to be at low levels. Higher mass fractions of As were observed in wetlands dominated by *T*. *chinensis* [26] and *P. australis* [9] in the YRD. As for Cd, our results were in the same order of magnitude as those reported in the YRD but far below those in *S. alterniflora* wetland in Bohai Bay [30]. Pb levels in the present study tended to be at a moderate level among those studies conducted in the YRD. Therefore, taking it by and large, the heavy metals in sediments in the *S. alterniflora* communities in our study were consistent with the major observations of metals conducted in different types of sediments of the YRD.

As for the spatial distribution issues, Co levels in Zone A were higher than those in Zone B (*p* < 0.05) and Zone C (*p* < 0.01). Similar observations were also made for the mass fractions of Ni and As. The mass fractions of Cd in Zone A were significantly higher than those in Zone C (*p* < 0.01). The Pb levels among the different zones showed no significant variance. So sediments in Kenli Country (Zone A and B) had more Co, Ni, As and Cd than those in Lijin Country. It was worth mentioning that Zone A and Zone B were adjacent to a harbor named Guangli Harbor, and related shipping and some other activities might be responsible for the relatively high levels of heavy metals. On the other hand, levels of Co and Pb in the *S. alterniflora* communities were significantly higher than those in the *S. heteroptera* community (*p* < 0.05). Thus, vegetation might be an important factor influencing the distribution of heavy metals in the wetlands of the YRD [31,32,33,34].

**Table 2:** Heavy metal mass fractions in sediments from the Yellow River Delta and some other regions (mg/kg).

Region	Type/Species	Co	Ni	As	Cd	Pb	Reference
YRD, China	*S. alterniflora*	9–16	14–28	3–14	0.08–0.24	18–37	This study
YRD, China	*Phragmites australis*	9–11	28–31	15–33	0.26–0.77	21–40	[9]
YRD, China	not mentioned		24–30	7-9	0.09–0.12	20–21	[13]
Shandong Peninsula	marine sediment			7–16	0.05–0.13	24–45	[15]
YRD, China	*Tamarix Chinensis*		36	31	0.68	21	[26]
Luoyuan wetland, China	*S. Alterniflora,* tideland	22–26	39–46	7–15	0.06–0.23	16–26	[27]
Kerala, India	sediment in fishing zones	17–30	20–70		1.1–2.3	29–74	[28]
Yangtze River estuary, China	marine sediment		18–56		0.01–0.16	9–37	[29]
Bohai Bay, China	*S. alterniflora*				8.21	48.12	[30]
YRD, China	soil			5–12	0.08–0.28	13–39	[31]
YRD, China	mud flat & *S. heteroptera* et al.			7.9		16.2	[32]
YRD, China	mud flat, thin reed, *S. heteroptera* et al.		11–36	4–13	0.02–0.84	4–26	[33]
YRD, China	not mentioned		18-45	6-17	0.05–0.57	14–33	[34]
Pearl River Estuary, China	mangrove sediment			29	0.96	43	[35]
Alexandria Coast, Egypt	marine sediment		33		0.3	33	[36]

### 3.2. Correlation between Heavy Metals and Sediment Properties

Correlations among different metals and physicochemical factors are presented in Table 3. It was obvious that the correlations between all metal elements were highly significant (*p* < 0.01), suggesting they might have the same sources or similar environmental behaviors. Sediment properties and heavy metals might interact with each other [13]. Sediment physicochemical parameters, such as TOC and grain size, have frequently been investigated to explore their influences on the distribution of heavy metals because they could affect the mobility and bioavailability of some trace elements [12,13,14]. In the present study, TOC was significantly related with Co, Ni and Pb (*p* < 0.01) and weakly correlated with As (*p* < 0.05). Clay was significantly related with Co, Ni and Pb and weakly correlated with As and Cd (*p* < 0.05). However, pH, whose values were relatively stable, was not related to any metal element. Thus, TOC and Clay tended to be key physiochemical factors affecting the distribution of most of the metals in the sediments of the *S. alterniflora* wetland in the YRD. It was reported that *S. alterniflora* could promote the deposition of fine particulate matter, thus increasing the enrichment of heavy metals in wetlands [37]. In addition, it was also reported that the invasion of *S. alterniflora* could increase TOC and nitrogen and reduce the pH, bulk density and salinity of coastal sediments [38]. In this study, the clay contents in the *S. alterniflora* communities were significantly higher than those in the *S. heteroptera* community (*p* < 0.05) which might lead to the relatively greater mass fractions of Co and Pb.

**Figure 2:**
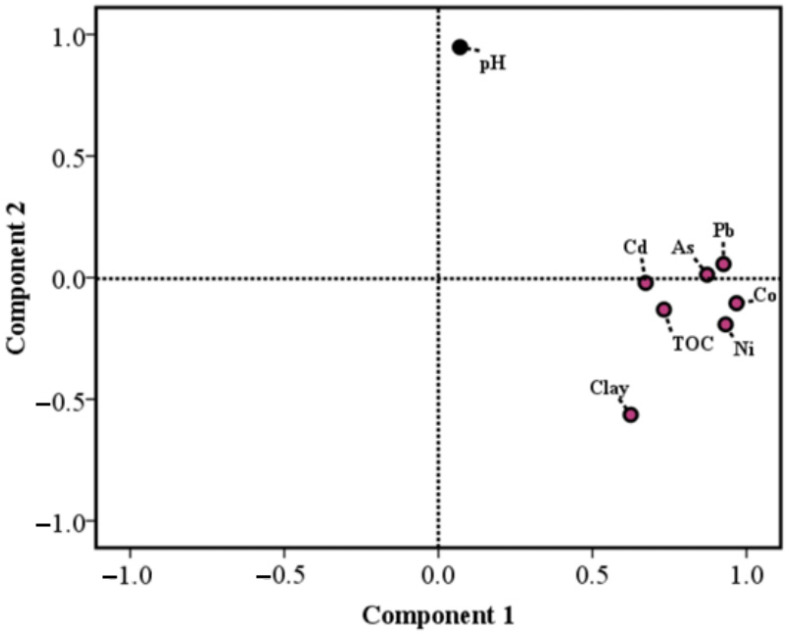
Factor analysis loading plot for *S. alterniflora* wetland in the Yellow River Delta.

PCA is commonly used to identify sources of metals [33,34]. In the present study, rotated component matrices are shown in Table 4. According to the results, in the *S. alterniflora* communities, the total variance in the heavy metal mass fractions could be explained by two factors that contributed 76.12% of the variance. All five metal elements, as well as TOC and Clay, dominated PC1, accounting for 61.35% of the total variance. PC2 explained 14.76% of the total variance, accompanied by great positive factor loadings for pH. Co, Ni, As, Cd and Pb had higher positive load values on PC1 and all the values were greater than 0.67, which was consistent with the significant correlation between those metals (Table 3). Co, Ni and Pb were observed with higher load values above 0.9, showing strong homology. These elements might mainly be from a lithogenic origin from the loess materials of the Loess Plateau accompanying transport by the Yellow River [26]. Besides, possible sources of them might include application of pesticides and fertilizers, oilfield development, waste water discharge, fishing and so on [33]. The factor loading plot indicated that the metals and physicochemical properties could be classified into two groups (Figure 2). These observations also suggested that the target elements had same/similar sources and were positively affected by TOC and clay rather than pH. In the present study, pH was observed with a narrow range of values. This might suggest that this parameter was influenced by natural processes, leading to the special factor loading of pH in PC2. As mentioned above, the mass fractions of the metals in our study were in accordance with the associated background values, and spatial variation was observed. Thus, in our opinion, PC1 could be explained by natural and anthropogenic factors, such as geogenic and/or sedimentation processes.

### 3.3. Risk Assessment

**Figure 3:**
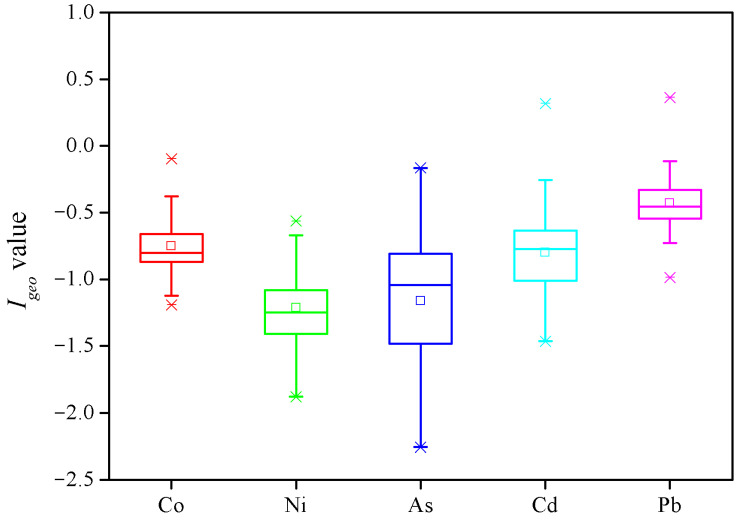
Geoaccumulation index for metal elements in sediments from the *S. alterniflora* wetland in the Yellow River Delta.

The *I_geo_* values of the metal elements in the wetlands of the YRD are presented in Figure 3. By comparison with the average values, an order of Pb (−0.37) > Co (−0.71) > Cd (−0.74) > As (−1.12) > Ni (−1.17) was observed. Those of Co, Ni and As were all below 0, indicating no contamination of these metals. In most of the sediment samples, *I_geo_* values of Cd and Pb were lower than 0, also presenting an unpolluted status. Only 1 for Cd and 4 for Pb were between 0 and 0.5, meaning that a few sampling sites were slightly polluted by Cd and Pb. Therefore, the contamination of heavy metals in the sediments tended to be negligible, considering their relatively small *I_geo_* values. Little *I_geo_* values lower than 0 were also reported in different types of wetlands in the YRD [21].

**Figure 4:**
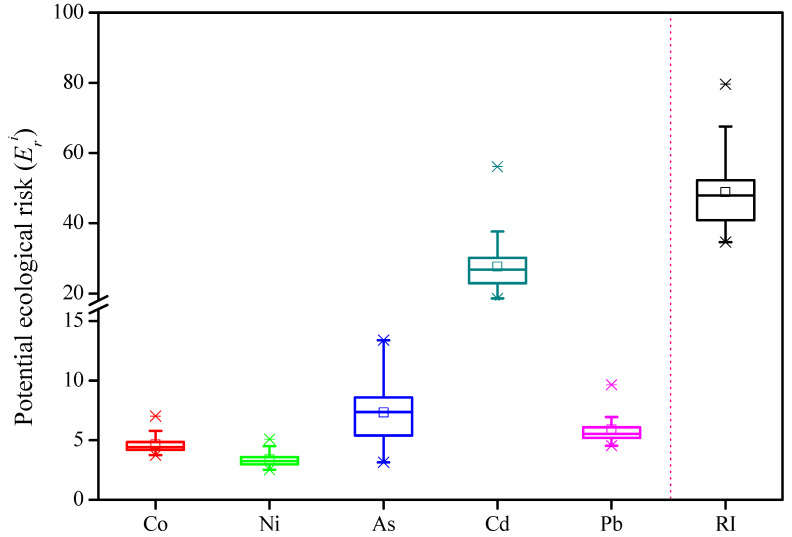
Box plot of potential ecological risk indexes for heavy metals in the *S. alterniflora* wetland in the Yellow River Delta.

The values of *E_r_^i^* and *RI* are shown in Figure 4. In detail, *E_r_^i^* values of Co, Ni, As, Cd and Pb ranged from 3.73 to 7.03, 2.52–5.08, 3.14–13.38, 18.59–56.14 and 4.53–9.65, respectively. It was obvious that a general order of Cd > As > Pb > Co > Ni was appropriate for most of the sampling sites. Co, Ni, As and Pb in sediments from the wetland were observed with low ecological risks according to their *E_r_^i^* values significantly lower than 40. In most of the sampling stations (>95%), the potential ecological risks of Cd were also slight (<40). Only one site was observed with a moderate risk of Cd in the sediment. In summary, the potential ecological risks caused by a single metal element in the sediments were very low.

The *RI* values of multiple metals in sediments of the *S. alterniflora* communities’ wetlands ranged from 34.66 to 79.61, with an average of 48.92 indicating that only 1 grade of ecological risks was posed to the aquatic environment by those elements. All *RI* values were lower than 150, suggesting very low risks. In addition, Cd was the major contributor (51–71%) to the *RI* values in all the samples due to its great environmental harmfulness. This coincided with many other studies conducted in areas/regions such as Shantou Bay [39] and Coastal Pearl Bay [40]. As for the spatial variation, an order of Zone A (54.36) > Zone B (48.44) > Zone C (39.90) was observed, revealing that relatively high ecological risks might be posed in those wetlands in Kenli Country (*p* < 0.01). In conclusion, the potential ecological risks caused by the five metals were at a slight level.

## 4. Conclusions

The present study investigated the mass fractions and distribution of heavy metals in surface sediments from *S. alterniflora* wetlands in the YRD. The results demonstrated that the wetlands were slightly polluted by trace metals. Due to anthropogenic and vegetation factors, spatial variations in metal distribution could be observed. The investigated metals seemed to have the same sources, and TOC and clay were essential factors affecting the occurrence of metals in sediments. The values of *I_geo_* of the investigated elements revealed a slight pollution status of these metals. According to the values of *E_r_^i^* and *RI*, the potential ecological risks of the five metals were low. These findings could reveal the achievements of the environmental protection measures taken in the Yellow River Delta.

## Figures and Tables

**Table 1:** Mass fractions (mg/kg) of selected metals in sediments from different wetland swamps in the Yellow River Delta.

Sampling Region		Co	Ni	As	Cd	Pb	pH	TOC (‰)	Clay (%)
A (*n* = 16)	minimum	8.5	13.9	3.8	0.08	17.6	6.8	4.6	1.0
	maximum	16.0	27.9	13.8	0.24	37.5	7.9	55.0	42.5
	mean	11.6	20.7	9.4	0.13	24.4	7.3	31.7	17.4
	median	11.2	20.8	9.2	0.12	23.1	7.7	42.4	17.0
	standard deviation	3.1	5.7	4.1	0.07	8.4	0.3	21.1	12.9
B (*n* = 12)	minimum	9.1	14.5	6.3	0.09	19.9	7.0	4.2	5.4
	maximum	11.2	19.8	8.8	0.15	26.9	8.0	7.5	20.6
	mean	9.9	17.7	7.5	0.12	22.5	7.5	5.5	13.0
	median	9.7	17.8	7.6	0.12	22.2	7.5	5.4	11.6
	standard deviation	0.9	2.2	1.0	0.02	2.91	0.4	1.2	4.5
C (*n* = 9)	minimum	9.2	14.6	3.2	0.08	19.7	7.3	0.7	11.5
	maximum	10.8	19.1	5.6	0.14	21.7	7.6	19.6	22.6
	mean	9.7	16.1	4.3	0.10	20.5	7.5	12.0	15.2
	median	9.6	15.8	4.0	0.10	20.4	7.5	11.9	14.9
	standard deviation	0.7	1.9	1.0	0.02	0.9	0.4	1.2	4.7
K (*n* = 6)	minimum	7.5	11.2	5.3	0.07	14.7	6.7	4.1	0.9
	maximum	7.9	15.5	5.8	0.08	15.1	7.8	5.6	7.3
	mean	7.7	13.8	5.6	0.07	14.9	6.7	4.9	4.6
	median	7.7	14.3	5.6	0.07	14.9	7.0	5.0	5.2
	standard deviation	0.2	1.8	0.2	<0.01	0.1	0.1	5.9	3.9

**Table 3:** Spearman’s correlation coefficient with a two-tailed test between the parameters of sediments in *S. alterniflora* swamps of the Yellow River Delta.

	Co	Ni	As	Cd	Pb	pH	TOC	Clay
Co	1							
Ni	0.934 **	1						
As	0.802 **	0.830 **	1					
Cd	0.576 **	0.651 **	0.594 **	1				
Pb	0.912 **	0.803 **	0.735 **	0.559 **	1			
pH	−0.029	−0.133	−0.021	−0.048	0.125	1		
TOC	0.728 **	0.635 **	0.602 *	0.213	0.624 **	−0.034	1	
Clay	0.661 **	0.640 **	0.386 *	0.353 *	0.596 **	−0.331 *	0.522 **	1

** Representing correlation is significant at *p* < 0.01. * Representing correlation is significant at *p* < 0.05.

**Table 4:** Variances and rotated component matrices of heavy metals and physicochemical parameters in sediments.

Principle Component	Initial Eigenvalues			Element	Sum of Squared Loadings			Factor Loading	
	Total	% Variance	Cumulative %		Total	% Variance	Cumulative %	PC1	PC2
1	4.908	61.352	61.352	Co	4.908	61.352	61.352	0.967	−0.105
2	1.181	14.763	76.116	Ni	1.181	14.763	76.116	0.932	−0.192
3	0.829	10.365	86.481	As				0.872	0.012
4	0.503	6.288	92.769	Cd				0.673	−0.022
5	0.259	3.243	96.012	Pb				0.925	0.056
6	0.165	2.057	98.069	pH				0.070	0.948
7	0.129	1.608	99.677	TOC				0.732	−0.132
8	0.026	0.323	100.000	Clay				0.624	−0.564

## Data Availability

Not applicable.
